# Metabolomic profiling of ascending thoracic aortic aneurysms and dissections - Implications for pathophysiology and biomarker discovery

**DOI:** 10.1371/journal.pone.0176727

**Published:** 2017-05-03

**Authors:** Christian Doppler, Kathrin Arnhard, Julia Dumfarth, Katharina Heinz, Barbara Messner, Christian Stern, Therese Koal, Kristaps Klavins, Katarina Danzl, Florian Pitterl, Michael Grimm, Herbert Oberacher, David Bernhard

**Affiliations:** 1 Cardiac Surgery Research Laboratory, University Clinic for Cardiac Surgery, Innsbruck Medical University, Innsbruck, Austria; 2 Cardiac, Vascular, and Thoracic Surgery, Medical Faculty, Johannes-Kepler University, Linz, Austria; 3 Institute of Legal Medicine and Core Facility Metabolomics, Innsbruck Medical University, Innsbruck, Austria; 4 Department of Cardiac Surgery, Vienna Medical University, Vienna, Austria; 5 Biocrates Life Sciences AG, Innsbruck, Austria; Universiteit van Amsterdam, NETHERLANDS

## Abstract

**Objective:**

Our basic understanding of ascending thoracic aortic aneurysm (ATAA) pathogenesis is still very limited, hampering early diagnosis, risk prediction, and development of treatment options. “Omics”-technologies, ideal to reveal tissue alterations from the normal physiological state due to disease have hardly been applied in the field. Using a metabolomic approach, with this study the authors seek to define tissue differences between controls and various forms of ATAAs.

**Methods:**

Using a targeted FIA-MS/MS metabolomics approach, we analysed and compared the metabolic profiles of ascending thoracic aortic wall tissue of age-matched controls (n = 8), bicuspid aortic valve-associated aneurysms (BAV-A; n = 9), tricuspid aortic valve-associated aneurysms (TAV-A; n = 14), and tricuspid aortic valve-associated aortic dissections (TAV-Diss; n = 6).

**Results:**

With sphingomyelin (SM) (OH) C22:2, SM C18:1, SM C22:1, and SM C24:1 only 4 out of 92 detectable metabolites differed significantly between controls and BAV-A samples. Between controls and TAV-Diss samples only phosphatidylcholine (PC) ae C32:1 differed. Importantly, our analyses revealed a general increase in the amount of total sphingomyelin levels in BAV-A and TAV-Diss samples compared to controls.

**Conclusions:**

Significantly increased levels of sphingomyelins in BAV-A and TAV-Diss samples compared to controls may argue for a repression of sphingomyelinase activity and the sphingomyelinase-ceramide pathway, which may result in an inhibition of tissue regeneration; a potential basis for disease initiation and progression.

## Introduction

Aneurysms in general represent a *Damocles sword* in the classical sense for patients diagnosed with one of these diseases, and a hidden version of the sword for the general population. As aneurysms usually occur in the absence of (clear) symptoms, the first detection is often a matter of chance, e.g. as a result of clinical imaging. Due to the devastating consequences of aneurysm-associated arterial dissections and ruptures there is significant interest in the development of tests for early diagnosis. In parallel, also the pathogenesis of many forms of aneurysms is not clear, which has so far hampered personalization of aneurysm treatment. The general wish is to better define the individual time-point when surgical intervention is indicated, and even more, to have specific non-surgical options to slow down or stop disease progression. Both, early diagnosis and improved treatment need a better understanding of these diseases.

In the past decade the effort to understand the pathogenesis of aneurysms has increased significantly. This knowledge has led to important progress in experimental treatment, early diagnosis, and risk assessment. Despite the progress made, our understanding of the pathogenesis of aneurysms in general and the ascending thoracic aortic aneurysm (ATAA), which make up 51% of all thoracic aortic aneurysms (TAA) [1], in particular, is still poor. Previously, several familial and syndromic genetic factors, associated with slight to very high risk for the development of ATAA, and their genetic basis were defined [2–7]. Importantly, the majority of ATAAs is of unknown origin [2, 8].

The major risk factor for non-syndromic ATAA formation is hypertension, and anti-hypertensive therapy is a *gold standard* in the treatment of ATAA patients. Whereas smoking, atherosclerosis, and inflammation have an influence on abdominal aortic aneurysm (AAA) formation, the role of these risk factors in ATAA formation is much less clear [9, 10].

The central histological findings in non-syndromic ATAA analyses are the loss of smooth muscle cells (media degeneration) and the alteration of elastic fibre structures [11, 12]. Importantly, older but also very new results suggest that even these two “hallmarks” of ATAA pathogenesis may not occur generally, and (non-syndromic) ATAA pathogenesis concepts may have to be revisited [13, 14]. Most factors analysed so far and thought to have an impact on ATAA formation originate from previous studies on AAA patient samples and from the broader field of cardiovascular research. Major factors are: the MMP2/9-TIMP system, smooth muscle stress and cell death, aging processes (telomere length), alterations in genes and protein expression and function (e.g. SOD, AKT, SMAD, osteopontin, angiotensin), and the factors mentioned above [2, 6, 7, 15].

Despite the discovery of a significant number of potentially pathogenesis-relevant factors, up to date, reliable clinical markers for the early detection, determination of disease stages and progression of ATAAs are totally absent. The “omics” technologies in general offer great opportunities for the discovery of pathophysiologically relevant factors usable as disease markers and markers for an improved stratification of diseases. These markers may also indicate disease-dependent pathological processes and serve as therapeutic targets in the future. Generally, despite great progress in recent years, this area is still at its beginning. The “omics” era in aneurysm research started in 1999 with a SAGE analysis of intracranial aneurysm [16], followed by the first transcriptome analyses in AAA tissue. Tung et al. revealed 44 genes that were differentially expressed between controls and AAA and suggested a pro-inflammatory, matrix degrading, pro-atherosclerotic, and smooth muscle cell depleting profile [17]. The first study on aneurysm proteome was published in 2007 and compared the proteome of ATAA samples from patients with tricuspid aortic valves and bicuspid aortic valves [18]. Up to date there are only 4 publications dealing with the metabolome of aortic aneurysms, and all have been done on AAA [19–22]. Ciborowski et al. could reveal increased levels of the lipid class of lysophosphatidylcholines in the secretome of *in vitro* incubated AAA tissue compared to controls. Further the authors describe alterations in amino acid metabolism and suggest that the analysis of enzymes relevant to catabolism and anabolism may reveal novel insights [23]. In another study by the same team, Ciborowski et al. demonstrate, that plasma metabolomic analyses has the potential to predict AAA disease stages [20]. In order to also investigate the role of metabolomic alterations in the ATAA we herewith conducted, to our knowledge, the first metabolomic analysis in samples of ATAA patients.

## Material and methods

### Ethics statement

This study has been approved by the Ethics Committee of the Medical University of Innsbruck and the Ethics Committee of the Medical University of Vienna and conforms to the Declaration of Helsinki. Written informed consent was obtained from all study participants.

### Patients

For this study ascending thoracic aortic tissue samples from 37 individuals were subjected to metabolomic profiling. The control group consisted of 8 samples, which stem from organ donors or heart transplant recipients. The presence of aneurysms, atherosclerotic or valvular diseases was excluded in the control group. 9 samples stem from BAV-A, 14 samples stem from TAV-A, and 6 samples stem from TAV-Diss, all of which underwent surgery of the ascending thoracic aorta. The 37 samples (out of samples collected from 220 individuals) analyzed in the course of this study were chosen after matching for age and sex and by excluding samples from patients suffering from inflammatory aortic disease and systemic connective tissue disorder.

### Sample collection and preparation

Following surgical extraction, cleaning, and rinsing of ascending thoracic aortic tissue samples, samples were immediately snap frozen in liquid nitrogen and stored at -80°C until further use. For metabolite extraction, per sample 40 mg of aortic tissue were placed in sterile polystyrene tube on dry ice, followed by adding 6 μl ice cold 100% ethanol (ultrapure; Carl Roth, Karlsruhe, Germany) per mg of tissue. Consequently, a 5 mm diameter hardened steel ball (Retsch, Haan, Germany) was added into the tubes, and tubes were placed in a Mixer Mill MM400 (Retsch, Haan, Germany), followed by breaking up of tissue pieces by applying a vibrational frequency of 30 Hz for 3 minutes. In the next step samples were sonicated (15 intervals, cycles: 10%, power: MS72/D; Sonopuls HD 200, Bandelin, Berlin, Germany) and by a centrifugation step (10 min/20,800 x g/4°C) all solid components were separated. Supernatants were collected and stored on dry ice until metabolite analyses.

### Analysis of amino acids, acylcarnitines, hexose, sphingomyelins, phosphatidylcholines and lysophosphatidylcholines

Following extraction, 20 μl of tissue homogenate supernatant was subjected to metabolite quantification using AbsoluteIDQ^®^ p150 kit (BIOCRATES Life Sciences AG, Innsbruck, Austria). For the complete analytical procedure see patent US 2007/0004044 (at http://www.freepatentsonline.com/20070004044.html), or manufacturers`instructions. Briefly, the assay system used allows for the simultaneous quantification of 163 metabolites from four different compound classes, i.e. 40 acylcarnitines (Cx:y), hydroxylacylcarnitines (C(OH)x:y), and dicarboxylacylcarnitines (Cx:y-DC), hexose (H1), 14 amino acids, 15 sphingomyelins (SMx:y) and N-hydroxylacyloylsphingosyl-phosphocholine (SM (OH)x:y), 77 phosphatidylcholines (PC, aa = diacyl, ae = acyl-alkyl) and 15 lysophosphatidylcholines. The targeted screening for these metabolites was performed by applying flow injection analysis with tandem mass spectrometric detection (FIA-MS/MS) acquisition with multiple reaction monitoring (MRM) in positive and negative ion mode. Quantification was achieved using internal standards.

The samples were analysed on a Knauer K-1001 LC pump (Knauer, Berlin, Germany) equipped with a CTC-PAL HTS9 autosampler (CTC Analytics AG, Zwingen, Switzerland) and hyphenated to a QTrap 3200 mass spectrometer (ABSciex, Toronto, Canada). Metabolite concentrations (μM) were automatically calculated by the MetIDQ^®^ software package, being a part of the AbsoluteIDQ^®^ p150 kit. Metabolites which showed insufficient measurement stability for a reference sample, which was prepared four times spread along the plate (relative standard deviation of metabolite concentrations > 30%) or metabolites which did not exceed a signal intensity of a minimum of 500 counts, were excluded from the analysis. A total of 92 metabolites were quantified in the samples.

### Analysis of ceramides and sphingomyelins

An additional assay for the analysis of ceramides and sphingomyelins was employed to increase the coverage of lipids. To do so, 20 μL of sample aliquot, 50 μL of H2O, 330 μL of 0.01% BHT in methanol and 670 μL CHCl3 were combined in a glass vial. The vial was transferred to ultrasonic bath for 60 seconds. Then the mixture was vortexed for 30 seconds and incubated for 30 min at room temperature. 300 μL of water was added to induce the phase separation. After vortexing the mixture for 10 seconds, it was centrifuged for 10 min at 1800 g. The lower organic phase was collected and evaporated under nitrogen. Finally, the lipid extract was dissolved in 200 μL of hexane/isopropanol/100 mM ammonium acetate (40/50/10, v/v/v).

The quantitative analyses of ceramides and sphingomyelins were performed using FIA–MS/MS. The analyses were carried out on API 4000 QTRAP triple quadrupole MS/MS system (AB Sciex Deutschland GmbH, Darmstadt, Germany) coupled to an Agilent 1200 Series HPLC systems and CTC Pal auto sampler (CTC Analytics, Zwingen, Switzerland), all controlled by the Analyst 1.6 software (AB Sciex Deutschland GmbH, Darmstadt, Germany). The FIA–MS/MS analysis was performed using hexane/isopropanol/100 mM ammonium acetate (40/50/10, v/v/v) as mobile phase. 10 μL of the sample extract was injected and the following flow gradient was applied: 0.0–1.6 min, 30 μL/min; 1.6–2.4 min, 30–200 μL/min; 2.4–2.5 min, 200–1500 μL/min; 2.5–4.4 min, 1500 μL/min; 4.4–4.5 min, 30 μL/min. MS/MS detection of ceramides and sphingomyelins were performed in negative electrospray ionization mode using multi-reaction monitoring (MRM). The ion source parameters were set as follows: curtain gas, 10 arbitrary units; ion source gas 1, 15 arbitrary units, ion source gas 2, 50 arbitrary units; ion source temperature, 250°C; ion spray voltage, -4500 V; collision gas pressure was set to medium. The dwell time for every MRM was set to 25 msec. Data evaluation and quantification of lipids were carried out using the MetIDQTM software (Biocrates Life Sciences AG, Innsbruck, Austria). Seven point calibration curve and internal standardization with isotopically labelled substances was used for the calculation of the lipid concentration. Note that employed analytical tools detect lipids as sum signals of several possible isobaric lipid species.

### Data handling

**Fig 1:**
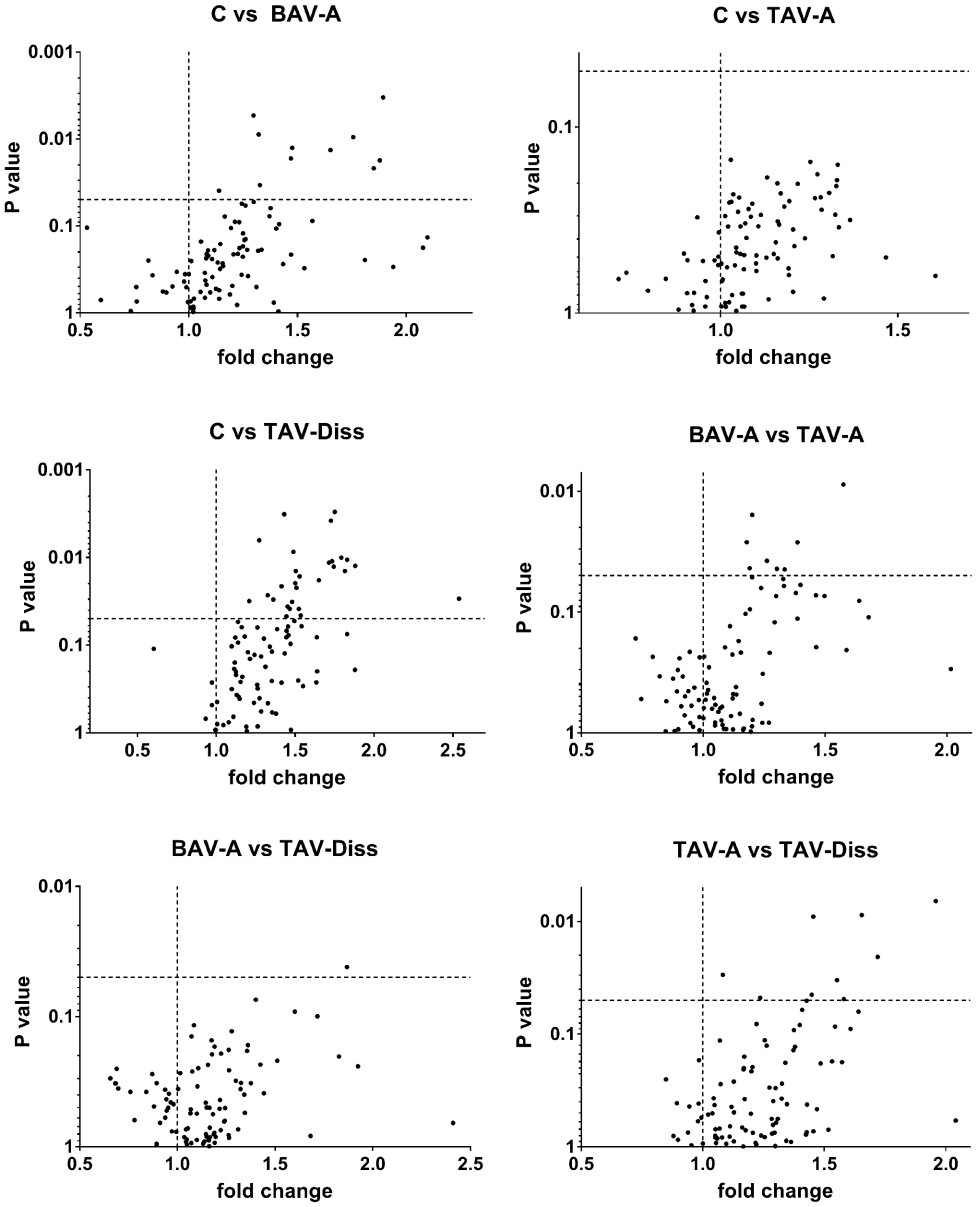
Volcano blot analyses reveal significant shifts in metabolite concentrations between controls and ATAA groups. The volcano blots shown in Fig 1 depict the differences between metabolite concentrations between the two groups indicated at the top of each blot. Each dot represents a single metabolite (out of the 92 detectable). On the x-axis the fold change is given (i.e. value (ratio) of the group named second, when group named first is set to one (dashed vertical line)).Values above p = 0.05 (dashed horizontal line) are considered to differ significantly between the two groups. C, control; BAV-A, bicuspid aortic valve-associated thoracic aneurysm; TAV-A, tricuspid aortic valve-associated thoracic aneurysm; TAV-Diss, tricuspid aortic valve-associated thoracic dissection.

**Fig 2:**
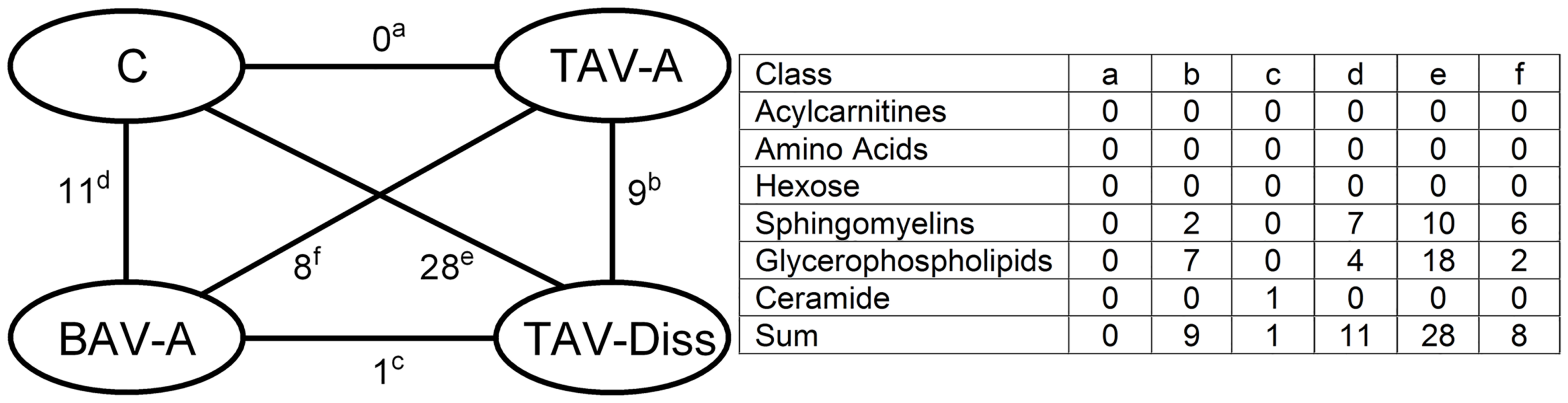
Differences in metabolites and metabolite compound classes between controls and ATAA forms. In the left side of Fig 2 the numbers near the lines which connect group symbols indicate the number of metabolites which differ significantly between groups. C, control (n = 8); BAV-A, bicuspid aortic valve-associated aneurysm (n = 9); TAV-A, tricuspid aortic valve-associated aneurysm (n = 14); TAV-Diss, tricuspid aortic valve-associated dissection (n = 6). Using the letters (a–f) superscripted to the numbers on the left side of Fig 2, metabolites which differ between groups can be assigned to compound classes in the table on the right side of the Figure. Significant differences (p<0.05) in individual metabolite concentration between groups were determined by ANOVA and multiple two-sided t-test comparisons (non-adjusted for maximum sensitivity).

**Fig 3:**
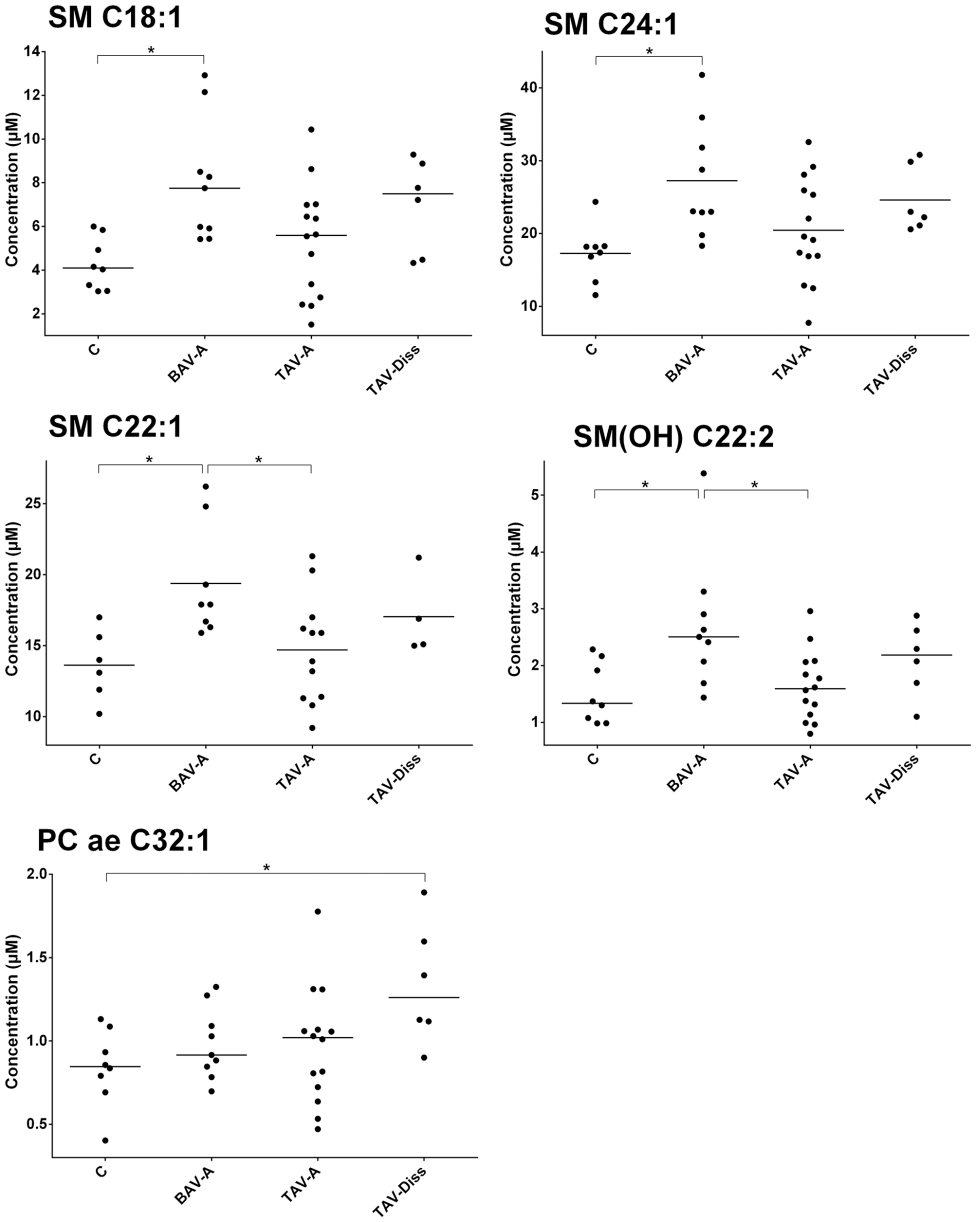
Metabolites with significant differences between controls and ATAA groups. Fig 3 shows single metabolites with significant differences in tissue concentration between groups (ANOVA, and multiple two-sided t-test comparisons, Bonferroni adjusted). C, control (n = 8); BAV-A, bicuspid aortic valve-associated aneurysm (n = 9); TAV-A, tricuspid aortic valve-associated aneurysm (n = 14); TAV-Diss, tricuspid aortic valve-associated dissection (n = 6). *, p<0.05. SM: sphingomyelin; PC ae: phosphatidylcholine acyl-alkyl.

**Fig 4:**
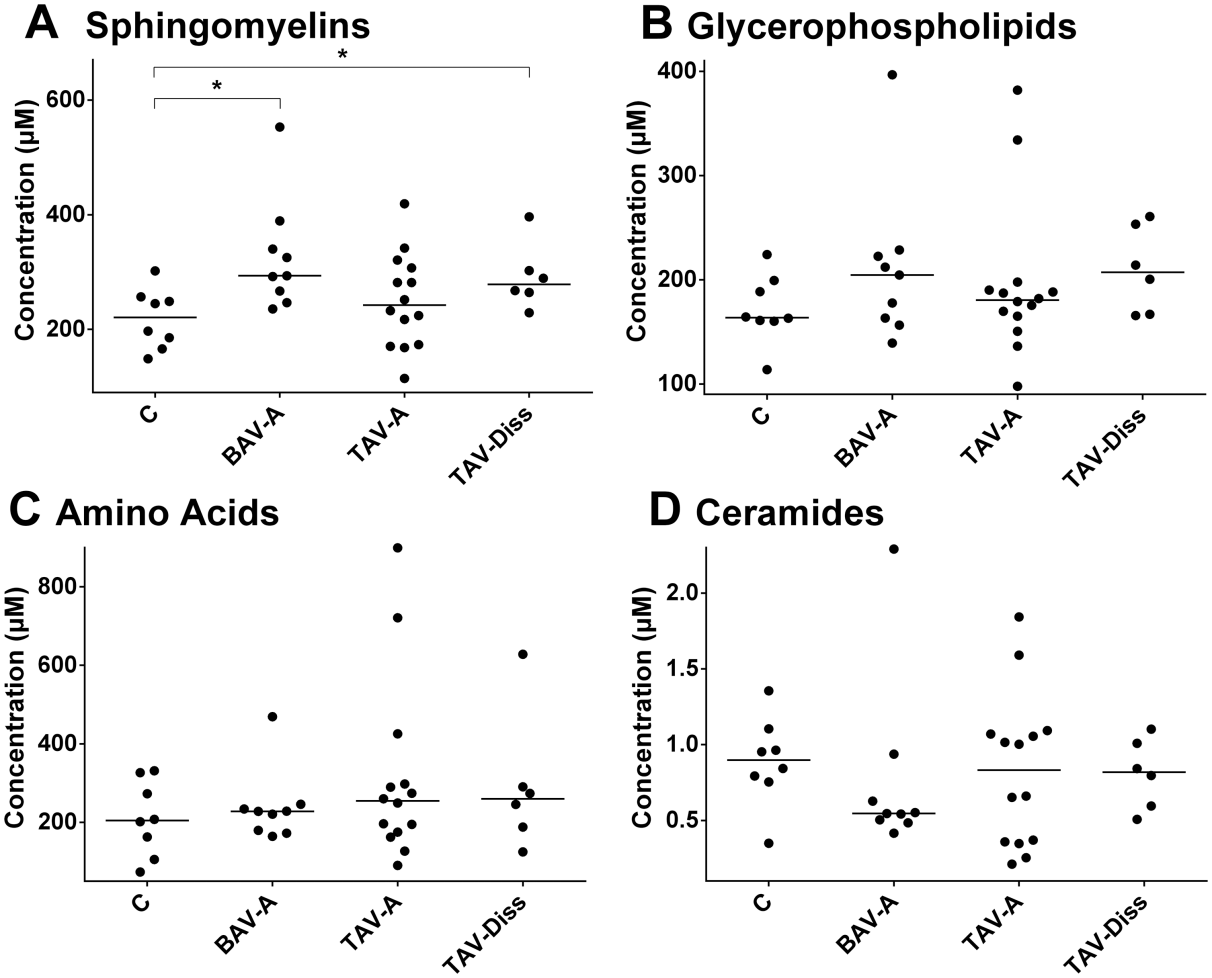
Total metabolite class concentrations in controls and different ATAA samples. Fig 4 shows the disease group-specific concentration of the sum of metabolites per metabolite class. The sums given include only those metabolites per class that were included in the analysis (for details see Table A in S1 File). C, control (n = 8); BAV-A, bicuspid aortic valve-associated aneurysm (n = 9); TAV-A, tricuspid aortic valve-associated aneurysm (n = 14); TAV-Diss, tricuspid aortic valve-associated dissection (n = 6). Dots represent median values per patient sample; black line indicates the groups mean value. Asterisks indicate significant differences between groups (ANOVA, Bonferroni adjusted) *, p<0.05.

**Fig 5:**
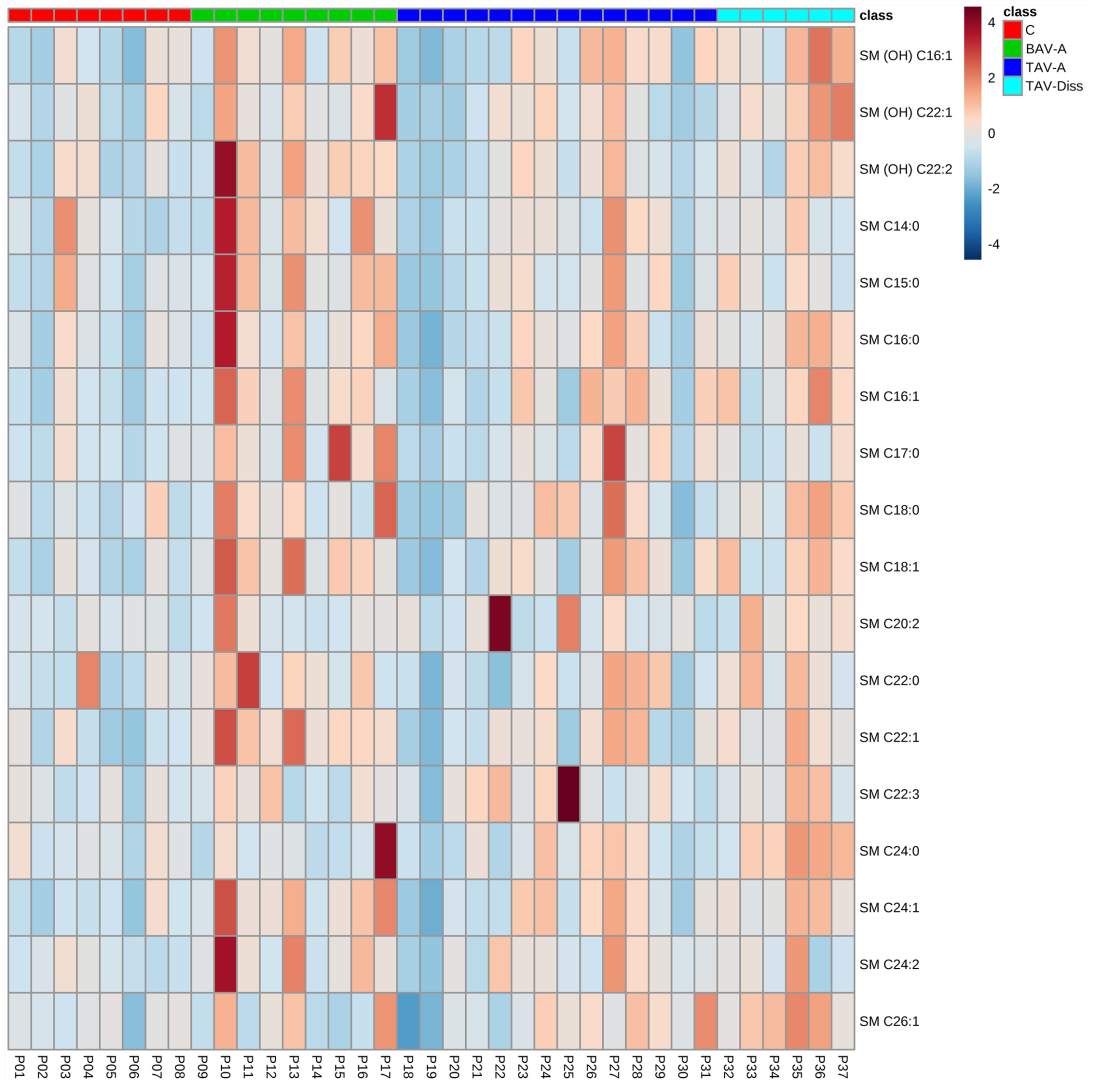
Hierarchical cluster analysis of total sphingomyelin concentrations. Fig 5 shows the distribution of sphingomyelin concentrations per patient tissue sample by hierarchical cluster analysis. Metabolite identity is indicted on the right side. Colours indicate compound concentrations. Colour scale bar (low concentration (dark blue) to high concentration (dark red) and group colour code are given in the upper right. C, control; BAV-A, bicuspid aortic valve-associated thoracic aneurysm; TAV-A, tricuspid aortic valve-associated thoracic aneurysm; TAV-Diss, tricuspid aortic valve-associated thoracic dissection; SM, sphingomyelin.

For the analysis and comparison of data of this study, different tools of analysis and different levels of data resolution were chosen. Generally, patients were subdivided into the following groups: controls, BAV-A, TAV-A and TAV-Diss, and metabolites between groups were compared. Fig 1 shows volcano blots which provide the ratio of the median concentration of all individual metabolites between two groups. The data from Fig 1 are summarized and ascribed to compound classed in Fig 2. A comparison of the groups, based on individual metabolites found to differ significantly between groups (Bonferroni corrected) is given in Fig 3. The tissue content of different metabolite substance classes (i.e. sphingomyelins, glycerophospholipids, amino acids, and ceramides) was calculated by summing up the concentrations of the individual metabolites per compound class per patient. Consequently these sums were then compared between the groups and are given in Fig 4. A single patient-based heatmap analysis of sphingomyelins is provided in Fig 5.

All primary values and data of this study are provided in the online supplement Table A in S1 File and Figures A and B in S1 File.

### Statistical analyses

All experimental data were analysed using SPSS statistics 22 software (IBM, Armonk, NY, USA). Basically, prior to further statistical analyses, all data were tested for a Gaussian distribution using the Kolmogorov-Smirnov test. Data with a Gaussian distribution (all data) were consequently analysed using one-way ANOVA and two-sided t-test comparisons. Multiple two-sided t-test comparisons were done using Bonferroni adjustment, with the exception of the data presented in Figs 1 and 2, where a maximum sensitivity was desired. Data shown in Figs 3 and 4 are individual values (dots) and the median (line). Groups were defined to differ significantly at a p value < 0.05. Figs 1, 3 and 4 were generated using GraphPad Prims 7 software (GraphPad Software, Inc., La Jolla, CA, USA). For the presentation of data in Fig 5 a heatmap was generated using the open source software MetaboAnalyst 3.0 (http://www.metaboanalyst.ca/, Wishart Research Group, University of Alberta, USA) [24].

## Results

In the course of the present study aortic tissue samples of 220 patients were collected. For each study participant basic clinical parameters were assessed (for details see Table 1), and the concentrations of 92 metabolites per sample were determined. After adjusting for age and gender, study groups were formed out of the 37 participants. With the exception of the potential confounder “diabetes” no significant difference could be observed in the clinical parameters between the groups.

**Table 1:** Aortic sample donor characteristics.

	Control n = 8	BAV-A n = 9	TAV-A n = 14	TAV-Diss n = 6	*p*-value
Male gender	50.0%	77.8%	64.3%	83.3%	0.524
Age (years)	63.0 (24–69)	62.0 (48–77)	66.5 (27–75)	71.0 (54–78)	0.172
Aortic diameter	n.d.*	54.1±8.9^1^	53.9±5.3	48.6±4.0	0.254
BSA (m^2^)	1.9±0.2	1.9±0.3	2.1±0.3	1.9±0.2	0.287
BMI	24.5±3.4	26.7±5.4	29.7±4.7	26.8±5.0	0.115
Diabetes	50%	0%	23.1%^1^	0%	0.037
Hyperlipidemia	50%	62.5%^2^	46.2%^1^	33.3%	0.758
Hypertension	37.5%	77.8%	58.3%^3^	83.3%	0.236
CAD	50%	25%^2^	16.7%^3^	33.3%	0.442

Table 1 summarizes patient characteristics per disease group and controls. Age is given as median (minimum—maximum), aortic diameter, BSA, and BMI are given in mean values ± SD, all other clinical factors are given in percent. Groups were tested for potential differences using multiple two-sided t-tests (Bonferroni adjusted), and p-values are indicated in the right row. BSA: body surface area, BMI: body mass index, CAD: coronary artery disease.

*, occurrence of an aneurysm was excluded.

(^1^ data available for 13 patients,

^2^ data available for 8 patients,

^3^ data available for 12 patients,

n.d., no data available; for raw data see online supplement S2 File)

### Metabolic profiles reveal striking similarities and differences between controls, BAV-A, TAV-A, and TAV-Diss

To reveal potential differences between the metabolome of control aortas and ATAAs, the concentration of 182 metabolites per sample were determined by FIA-MS/MS. Of those 182 analytes, 92 were detectable (i.e. above the lower level of detection; LOD, see Table A in S1 File). The different groups (controls, BAV-A, TAV-A, and TAV-Diss) were then compared by non-adjusted two-sided independent samples two-sided t-tests and volcano blotting to get an overview of existing trends. By comparing the number of metabolites which differed between the groups striking similarities and differences were evident. Fig 1 shows that not a single metabolite (out of 92 detectable metabolites) differs between healthy aortic wall and TAV-A samples. Similarly, also BAV-A and TAV-Diss samples differed only in one metabolite on a significant level (p<0.05). On the contrary, the control group differed in 11 metabolites from the BAV-A group, and in 28 metabolites from the TAV-Diss group. The TAV-A group differed in 8 metabolites from the BAV-A group and in 9 metabolites from the TAV-Diss group. Overall, 38 metabolites were found to differ significantly (with the limitation of a non-adjusted comparison) between at least two groups (see Fig 2 and Table A in S1 File). Of note, all metabolites which showed significant differences between the groups stem from the metabolite classes of sphingomyelins, glycerophospholipids and ceramides (Fig 2).

### Single metabolites are of limited usefulness for the differentiation of ATAA groups and controls

To allow for a single metabolite-based precise and stricter differentiation between groups, multiple Bonferroni-adjusted two-sided t-test comparisons were performed. These analyses resulted in only five metabolites which differed significantly between at least two groups. These metabolites, shown in Fig 3 were: PC ae C32:1, SM (OH) C22:2, SM C18:1, SM C22:1, and SM C24:1. Unfortunately, none of these markers (including the combination of these markers) allowed for a clear cut differentiation between the disease types and controls, as the distribution of values showed significant overlaps between groups.

### Differences in aortic wall total sphingomyelin content suggest extended metabolic alterations in BAV-A and TAV-Diss

To compare the total status of substance groups (i.e. sphingomyelins, glycerophospholipids, amino acids, and ceramides), the absolute concentrations of all individual metabolites from the same compound class per sample were summarized and groups were compared. These analyses, shown in Fig 4, revealed that sphingomyelins are increased in BAV-A (*p* = 0.018) and TAV-Diss (*p* = 0.012) when compared to the control. Other group comparisons for sphingomyelins did not result in further differences however a trend between BAV-A vs TAV-A was indicated but did not differ significantly (*p* = 0.058). Fig 5 shows a heat map comparison of these data. As we could observe a significantly different proportion of individuals suffering from diabetes between the disease groups and controls (see Table 1), and to control for this potential confounding factor, the same analyses were performed, including only the non-diabetic individuals per disease group. The results showed that the mean value ratios between disease groups remained the same. However, probably due to the smaller number of samples per group, the difference did not reach significance. For details, also see Figure A in S1 File.

The sum of absolute values for all other compound classes (i.e. glycerophospholipids, amino acids, ceramides) did not reveal significant differences (see Fig 4 and Figure B jn S1 File).

## Discussion

The role of alterations in metabolism in general and lipid metabolism in particular, as a potential factor in aneurysm pathogenesis is hardly investigated. In order to address this issue, the current study is–to our knowledge–the first study analysing the metabolomic state of aneurysm tissue in an ATAA setting.

A central and surprising result of this study was the observation of close similarities between control and TAV-A tissue, with no single metabolite differing between these groups, even using multiple t-test comparisons without correction. Based on these results, and within the limits of our targeted-metabolomics approach, it may be speculated that TAV-A formation may not be related to the metabolic factors analysed in the aortic wall. On the contrary, BAV-A samples and TAV-Diss samples differed significantly from controls in their sphingomyelin content. A confounding role of diabetes in the observed similarities and differences between disease groups is suggested to be unlikely, as a repetition of all analyses in the non-diabetic individuals per disease group gave similar results.

Sphingomyelins and ceramides are connected via the so called “sphingomyelinase-ceramide pathway”, and ceramides are products of sphingomyelin hydrolysation by the enzyme sphingomyelinase [25]. Generally, the active sphingomyelinase-ceramide pathway is thought to exert pro-inflammatory, pro-oxidative, and cell death-inducing activities, resulting in atherosclerosis, aging and cardiovascular events [26–29]. Importantly, an active sphingomyelinase-ceramide pathway, characterised by increased levels of ceramides and metabolisation of sphingomyelins (e.g. by oxidised LDL-induced sphingomyelinase activity) leads to reduced levels of sphingomyelins [30–33]. The finding of this study, that sphingomyelins are increased in BAV-A and TAV-Diss samples suggests that these classical pro-atherogenic processes may not play a role in these forms of aortic diseases. As a matter of fact, in TAAs (in contrast to AAAs) atherosclerosis risk factors and processes do not seem to constitute central elements in pathogenesis [34]. Nevertheless, the finding of increased levels of sphingomyelins in BAV-A and TAV-Diss aortic samples, and reduced levels of ceramides in BAV-A samples (not significant) suggests that compared to controls (and TAV-A samples), sphingomyelinase activity is reduced/inhibited. Based on these results–even if speculative—we suggest that insufficient activity of the sphingomyelinase-ceramide pathway in BAV-A and TAV-Diss patients may lead to insufficient tissue repair and regeneration processes, both of which need cell death and mild pro-inflammatory stimuli. Of interest is a study by Gabandé-Rodríguez E et al. who reported that increased levels of sphingomyelins in the course of Niemann-Pick disease A (caused by mutations in the gene SMPD1 encoding for the acid sphingomyelinase [35]) lead to inefficient autophago–lysosomal clearance in neurons and fibroblasts. The inefficient autophago–lysosomal clearance leads to (pathologically) reduce degradation of cytoplasmic organelles and cytosolic components [36]. Chen Y. et al. reported on reduced proliferation of hepatocyte cell lines by increased sphingomyelin levels [37], which may also indicate reduced regenerative capabilities under conditions of increased sphingomyelin levels.

Another interesting result of our analyses is the obvious difference in the metabolome between TAV-A and TAV-Diss, as well as similarity between TAV-Diss and the BAV-A group. A higher risk of suffering from aortic dissections by BAV-A patients compared to TAV-A patients is controversially discussed [38, 39], though metabolites differing between controls and TAV-Diss as well as TAV-A and TAV-Diss may be related to the occurrence of a dissection. Without doubt, the differences between the groups observed may well be based on processes other than sphingomyelinase activity, e.g. increased activity of sphingomyelinsynthase and/or increased activity of ceramidase etc. [32, 33, 40]. In the absence of detailed enzyme activity profiles the above concept is based on the principal of Occam`s razor and clearly is not more than a hypothesis which needs further studies.

The second goal of this study was to define potential biomarkers for a later follow up serum study. The rationale of analysing aortic tissue samples was to reveal potential pathophysiological differences between the groups (see above), and second (for biomarker search), to reduce the signal-to-noise ratio by analysing the diseased tissue and not serum which mirrors the metabolome of the entire body. The basic hypothesis for the biomarker search was accordingly, to not focus on systemic factors, but to focus on local disease processes and markers in the aorta. In summary, the results show significant differences within aortic tissue that allow the differentiation of BAV-A and TAV-Diss from controls. A differentiation of TAV-A from controls was not possible. As above mentioned, the observation of increased levels of sphingomyelins in BAV-A and TAV-Diss shall form the basis for a deeper analysis of the enzyme network involved in sphingomyelin synthesis and degradation. A clearer view on this network may lead to novel biomarkers (e.g. enzyme expression/activity) and may also reveal novel pathophysiological processes and treatment targets.

Hammad et al. previously showed good detectability of sphingomyelins in human serum and plasma, whereby SM C16:0 and SM C24:1, were the predominant sphingomyelin species in healthy human serum [41]. In our tissue study these two metabolites were also the main component (44.4%), which makes us hope that the presented results on sphingomyelins can be translated into a serum-based assay. Even though our study shows that single metabolites cannot be used as biomarkers to differentiate between disease groups and controls, a combination of several markers i.e. the analysis of the concentration of an entire substance class may lead to more stable results. Also Ikonomidis et al. recently reported, that a successful differentiation of TAA samples and healthy controls could not be achieved by single proteins, but by a combination of several proteins, resulting in sensitive and specific results [42].

In this study we chose to stratify ATAAs and to differentiate between BAV-A, TAV-A, and TAV-Diss. Currently, among experts it is controversially discussed whether these potential subtypes of ATAAs reflect in fact subtypes of the same disease (same pathogenetic process) or are in fact different diseases with a similar outcome. There are a number of studies which counter-argue these differences, and could not reveal subtype specific characteristics between BAV-A and TAV-A, neither on the genomic nor the proteomic level [43, 44]. However, recent studies could provide new evidence for such a difference by analysing protein expressions in the plasma [42], and by detailed histopathological comparisons [45]. The results of the present study on aortic tissue is also in support for a difference in pathogenesis between TAV-A and BAV-A, and may add to the discussion by showing that TAV associated aneurysms and dissections may form two different disease entities.

### Study limitations

Due to the fact that this study uses aortic tissue instead of serum/plasma and also because of matching the groups by age etc., the group size is relatively small. Also the choice for a targeted metabolomics approach (chosen to know the identity of all analysed metabolites) limits the results and conclusions drawn to these metabolites. As mentioned, due to the analysis of tissue and not serum/plasma the applicability of the observed differences in the metabolomic profiles of groups in blood tests is uncertain, as different profiles of analytes in plasma and tissue were previously observed [42]. Further, by focusing on tissue we excluded certain systemic-driven aspects in disease pathogenesis. Nevertheless, as the aortic wall is the disease site, also systemic processes, relevant to disease metabolism shall be reflected by the aorta tissue.

Finally, it cannot be excluded that unknown confounders alter the metabolic profile of the individual groups and may be the basis for the observed differences (differences in patients’ medications, body composition etc.).

## Supporting information

S1 File **Table A–Metabolite concentrations and significant differences (non-adjusted) between the groups for all detectable metabolites. S**ummarizes the absolute concentrations of individual metabolites (2^nd^ to 5^th^ row from left), by metabolite class. Only metabolites above the lower level of detection and metabolites which differed significantly between at least two groups (ANOVA analyses and multiple t-tests (non-adjusted for maximum sensitivity)) are listed. Values are given as median ± SD (in μM). C, control; BAV-A, bicuspid aortic valve-associated thoracic aneurysm; TAV-A, tricuspid aortic valve-associated thoracic aneurysm; TAV-Diss, tricuspid aortic valve-associated thoracic dissection. In the right side of the table the difference between groups is given in %; the corresponding p-values are indicated. “+” prior to the values (in %) indicate that the first group shows higher concentrations compared to the second group. B-C, BAV-A versus C; D-C, TAV-Diss versus C; D-T, TAV-Diss versus TAV-A. Comparisons: TAV-A versus C is not given, because no significant differences between groups were observed. Empty fields indicate the absence of a significant difference. **Figure A- Total sphingomyelin concentrations in non-diabetic controls and different ATAA samples**—shows the disease group-specific concentration of the sum of metabolites for sphingomyelins. The sums given include only those metabolites per class that were included in the analysis (for details see Table A in S1 File). Patients suffering from diabetes were excluded from the analysis. C, control (n = 4); BAV-A, bicuspid aortic valve-associated aneurysm (n = 9); TAV-A, tricuspid aortic valve-associated aneurysm (n = 11); TAV-Diss, tricuspid aortic valve-associated dissection (n = 6). Dots represent median values per patient sample; black line indicates the groups mean value. Asterisks indicate significant differences between groups (ANOVA, Bonferroni adjusted) *, p<0.05. **Figure B—Hierarchical cluster analysis of total metabolite concentrations—**shows the distribution of total metabolite concentrations (i.e. sphingomyelins, glycerophospholipids, amino acids and ceramides) per patient tissue sample by hierarchical cluster analysis. Metabolite identity is indicted on the right side. Colors indicate compound concentrations. Color scale bar (low concentration (dark blue) to high concentration (dark red) and group color code are given in the upper right. C, control; BAV-A, bicuspid aortic valve-associated thoracic aneurysm; TAV-A, tricuspid aortic valve-associated thoracic aneurysm; TAV-Diss, tricuspid aortic valve-associated thoracic dissection.
(DOCX)

S2 File Raw data. (XLS)
