# Supplementary material for: Metabolomic profiling of ascending thoracic aortic aneurysms and dissections - Implications for pathophysiology and biomarker discovery
Source: PLoS One. 2017 May 3;12(5):e0176727. doi: 10.1371/journal.pone.0176727 (PMC5415060; doi:10.1371/journal.pone.0176727)
Supplement: S1 File — Table A–Metabolite concentrations and significant differences (non-adjusted) between the groups for all detectable metabolites. Summarizes the absolute concentrations of individual metabolites (2nd to 5th row from left), by metabolite class. Only metabolites above the lower level of detection and metabolites which differed significantly between at least two groups (ANOVA analyses and multiple t-tests (non-adjusted for maximum sensitivity)) are listed. Values are given as median ± SD (in μM). C, control; BAV-A, bicuspid aortic valve-associated thoracic aneurysm; TAV-A, tricuspid aortic valve-associated thoracic aneurysm; TAV-Diss, tricuspid aortic valve-associated thoracic dissection. In the right side of the table the difference between groups is given in %; the corresponding p-values are indicated. “+” prior to the values (in %) indicate that the first group shows higher concentrations compared to the second group. B-C, BAV-A versus C; D-C, TAV-Diss versus C; D-T, TAV-Diss versus TAV-A. Comparisons: TAV-A versus C is not given, because no significant differences between groups were observed. Empty fields indicate the absence of a significant difference. Figure A- Total sphingomyelin concentrations in non-diabetic controls and different ATAA samples—shows the disease group-specific concentration of the sum of metabolites for sphingomyelins. The sums given include only those metabolites per class that were included in the analysis (for details see Table A in S1 File). Patients suffering from diabetes were excluded from the analysis. C, control (n = 4); BAV-A, bicuspid aortic valve-associated aneurysm (n = 9); TAV-A, tricuspid aortic valve-associated aneurysm (n = 11); TAV-Diss, tricuspid aortic valve-associated dissection (n = 6). Dots represent median values per patient sample; black line indicates the groups mean value. Asterisks indicate significant differences between groups (ANOVA, Bonferroni adjusted) *, p<0.05. Figure B—Hierarchical cluster analysis of total m [file pone.0176727.s001.docx]

**S1 File**

**to**

**Metabolomic profiling of ascending thoracic aortic aneurysms and dissections**

**– implications for pathophysiology and biomarker discovery**

By Doppler et al.

**Table A– Metabolite concentrations and significant differences (non-adjusted) between the groups for all detectable metabolites**

| **Metabolites** | **C** | **BAV-A** | **TAV-A** | **TAV-Diss** | **Significant differences among groups %**  **(p-valve)** | | | | |
| --- | --- | --- | --- | --- | --- | --- | --- | --- | --- |
| **Sphingomyelins** | | | | | B-C | D-C | B-T | D-T | D-B |
| SM (OH) C16:1 | 3.411  ±1.238 | 5.033  ±1.145 | 4.223  ±1.795 | 5.951  ±1.818 | +67.8  (0.013) | +74.5  (0.013) |  |  |  |
| SM (OH) C22:1 | 1.280  ±0.444 | 1.577  ±0.911 | 1.182  ±0.557 | 1.956  ±0.705 |  | +52.8  (0.016) | +75.0  (0.044) | +65.5  (0.009) |  |
| SM (OH) C22:2 | 1.334  ±0.535 | 2.506  ±1.159 | 1.591  ±0.613 | 2.185  ±0.664 | +53.2  (0.018) |  | +63.5  (0.009) |  |  |
| SM C16:0 | 89.78  ±23.28 | 111.7  ±50.7 | 85.93  ±37.09 | 108.55±28.29 |  | +20.9  (0.031) |  |  |  |
| SM C16:1 | 11.48  ±4.19 | 20.16  ±9.11 | 14.53  ±8.95 | 21.58  ±8.49 | +57.0  (0.010) | +88.1  (0.012) |  |  |  |
| SM C18:0 | 9.31  ±1.63 | 10.85  ±3.5 | 10.25  ±3.34 | 12.35  ±2.43 |  | +32.6  (0.027) |  |  |  |
| SM C18:1 | 4.093  ±1.187 | 7.749  ±2.816 | 5.589  ±2.596 | 7.488  ±2.139 | +52.8  (0.003) | +82.9  (0.011) | +72.1  (0.026) |  |  |
| SM C20:2 | 0.475  ±0.079 | 0.480  ±0.259 | 0.481  ±0.427 | \| 0.647  ±0.202 \| \| --- \| |  | +36.2  (0.030) |  |  |  |
| SM C22:3 | \| 16.58  ±3.49 \| \| --- \| | 18.8  ±5.2 | 17.88  ±11.62 | 18.87  ±5.72 |  |  |  |  |  |
| SM C24:0 | 8.676  ±2.179 | 7.782  ±7.886 | 7.645  ±3.876 | 14.97  ±4.01 |  | +72.6  (0.004) |  | +95.7  (0.007) |  |
| SM C24:1 | 17.76  ±3.80 | 23.05  ±7.90 | 19.35  ±7.10 | 22.60  ±4.52 | +77.1  (0.006) | +27.2  (0.006) | +84.0  (0.043) |  |  |
| SM C26:1 | 0.326  ±0.061 | 0.272  ±0.127 | 0.327  ±0.129 | 0.467  ±0.096 |  | +43.3  (0.003) |  |  |  |
| SM C14:0 | 12.85  ±3.40 | 16.95  ±4.66 | 13.70  ±2.86 | 14.25  ±1.51 |  |  |  |  |  |
| SM C15:0 | 11.60  ±1.94 | 13.85  ±2.87 | 11.75  ±1.89 | 13.25  ±1.14 |  |  | +17.9  (0.026) |  |  |
| SM C17:0 | 11.45  ±1.46 | 14.25  ±4.18 | 11.55  ±3.84 | 12.15  ±1.29 | +24.5  (0.039) |  |  |  |  |
| SM C22:1 | 13.55  ±2.25 | 17.90  ±3.69 | 14.90  ±3.61 | 16.00  ±2.51 | +32.1  (0.009) |  | +20.1  (0.016) |  |  |
| SM C22:0 | 12.90  ±2.71 | 15.55  ±2.89 | 12.95  ±2.68 | 16.25  ±1.76 |  |  |  |  |  |
| SM C24:2 | 15.90  ±1.89 | 18.80  ±8.08 | 17.45  ±4.81 | 18.35  ±4.61 |  |  |  |  |  |
| **Glycerophospholipids** | | | | |  |  |  |  |  |
| lysoPC a C16:0 | 2.636  ±2.246 | \| 4.770  ±9.309 \| \| --- \| | 3.185  ±1.448 | 3.328  ±3.054 |  |  |  |  |  |
| lysoPC a C16:1 | 0.045  ±0.042 | 0.094  ±0.174 | 0.072  ±0.051 | 0.115  ±0.057 |  | +155.6  (0.029) |  | +59.7  (0.049) |  |
| lysoPC a C17:0 | 0.082  ±0.070 | 0.172  ±0.274 | 0.086  ±0.055 | 0.113  ±0.122 |  |  |  |  |  |
| lysoPC a C18:0 | 1.616  ±1.005 | 2.376  ±4.714 | 1.721  ±0.793 | 1.627  ±1.416 |  |  |  |  |  |
| lysoPC a C18:1 | 0.591  ±0.839 | 0.906  ±2.663 | 0.552  ±0.303 | 0.618  ±0.617 |  |  |  |  |  |
| lysoPC a C18:2 | 0.321  ±0.665 | 0.623  ±3.227 | 0.371  ±0.240 | 0.473  ±0.594 |  |  |  |  |  |
| lysoPC a C20:4 | 0.262  ±0.255 | 0.375  ±0.585 | 0.338  ±0.257 | 0.354  ±0.307 |  |  |  |  |  |
| lysoPC a C28:0 | 0.072  ±0.032 | 0.080  ±0.081 | 0.094  ±0.081 | 0.135  ±0.059 |  |  |  |  |  |
| PC aa C28:1 | 1.152  ±0.577 | 1.807  ±1.129 | 1.387  ±0.521 | 1.695  ±0.527 |  |  | +76.8  (0.044) |  |  |
| PC aa C30:0 | 1.931  ±0.760 | 2.450  ±0.777 | 2.556  ±1.058 | 2.613  ±1.020 |  |  |  |  |  |
| PC aa C32:0 | 5.217  ±1.809 | 6.201  ±2.772 | 6.920  ±6.060 | 7.589  ±3.050 |  |  |  |  |  |
| PC aa C32:1 | 1.675  ±0.476 | 2.196  ±0.513 | 2.152  ±1.213 | 1.933  ±0.821 |  |  |  |  |  |
| PC aa C32:2 | 0.643  ±0.142 | 0.743  ±0.369 | 0.682  ±0.241 | 0.938  ±0.247 |  |  |  |  |  |
| PC aa C34:1 | 33.61  ±6.87 | 37.82  ±7.85 | 30.43  ±12.06 | 33.80  ±4.49 |  |  |  |  |  |
| PC aa C34:2 | 14.02  ±3.82 | 16.23  ±7.68 | 13.95  ±10.55 | 17.02  ±5.46 |  |  |  |  |  |
| PC aa C34:3 | 0.556  ±0.137 | 0.817  ±0.307 | 0.708  ±0.295 | 0.953  ±0.196 | +68.0  (0.017) | +71.4  (0.011) |  |  |  |
| PC aa C34:4 | 0.106  ±0.027 | 0.138  ±0.038 | 0.133  ±0.091 | 0.174  ±0.073 |  |  |  |  |  |
| PC aa C36:0 | 0.365  ±0.296 | 0.508  ±0.155 | 0.347  ±0.192 | 0.597  ±0.132 |  |  |  | +72.0  (0.021) |  |
| PC aa C36:1 | 9.218  ±1.976 | 9.405  ±2.497 | 8.856  ±2.406 | 10.373  ±2.216 |  |  |  |  |  |
| PC aa C36:2 | 12.15  ±2.72 | 13.46  ±5.80 | 12.83  ±7.79 | 15.62  ±2.81 |  |  |  |  |  |
| PC aa C36:3 | 6.803  ±1.819 | 8.493  ±5.381 | 7.383  ±4.351 | 9.614  ±1.972 |  | +41.3  (0.021) |  |  |  |
| PC aa C36:4 | 12.96  ±3.19 | 13.70  ±4.65 | 15.04  ±9.98 | 15.05  ±5.00 |  |  |  |  |  |
| PC aa C36:5 | 0.680  ±0.192 | 0.849  ±0.232 | 0.757  ±0.392 | 1.190  ±0.211 |  | +75.0  (0.003) |  |  |  |
| PC aa C36:6 | 0.074  ±0.017 | 0.093  ±0.044 | 0.082  ±0.037 | 0.113  ±0.039 |  |  |  |  |  |
| PC aa C38:3 | 9.518  ±2.334 | 11.67  ±6.32 | 9.938  ±2.388 | 10.639  ±2.235 |  |  |  |  |  |
| PC aa C38:4 | 20.17  ±4.58 | 21.98  ±6.59 | 22.32  ±6.72 | 19.64  ±6.54 |  |  |  |  |  |
| PC aa C38:5 | 3.606  ±0.895 | 3.879  ±1.240 | 3.869  ±3.080 | 4.071  ±0.964 |  |  |  |  |  |
| PC aa C38:6 | 1.569  ±0.527 | 1.950  ±1.231 | 1.821  ±0.853 | 2.813  ±0.613 |  | +79.3  (0.010) |  |  |  |
| PC aa C40:4 | 1.875  ±0.608 | 1.920  ±0.551 | 1.902  ±0.491 | 2.059  ±0.758 |  |  |  | +8.3  (0.030) |  |
| PC aa C40:5 | 1.357  ±0.308 | 1.328  ±0.492 | 1.348  ±0.639 | 1.521  ±0.367 |  |  |  |  |  |
| PC aa C40:6 | 1.348  ±0.385 | 1.536  ±0.925 | 1.239  ±0.637 | 2.034  ±0.484 |  | +50.9  (0.022) |  |  |  |
| PC aa C42:2 | 0.036  ±0.010 | 0.036  ±0.011 | 0.041  ±0.026 | 0.055  ±0.018 |  | +52.8  (0.037) |  |  |  |
| PC aa C42:5 | 0.044  ±0.013 | 0.054  ±0.021 | 0.058  ±0.042 | 0.066  ±0.015 |  | +50.0  (0.020) |  |  |  |
| PC ae C30:1 | 0.262  ±0.136 | 0.485  ±0.169 | 0.384  ±0.149 | 0.379  ±0.156 | +54.0  (0.022) |  | +79.3  (0.038) |  |  |
| PC ae C32:1 | 0.846  ±0.230 | 0.916  ±0.215 | 1.019  ±0.348 | 1.260  ±0.364 |  | +48.9  (0.009) |  | +23.7  (0.047) |  |
| PC ae C32:2 | 0.638  ±0.227 | 1.054  ±0.374 | 0.721  ±0.409 | 1.159  ±0.371 | +60.6  0.013 | +81.7  (0.014) |  |  |  |
| PC ae C34:0 | 0.370  ±0.120 | 0.342  ±0.146 | 0.384  ±0.244 | 0.548  ±0.201 |  | +48.1  (0.032) |  |  |  |
| PC ae C34:1 | 1.596  ±0.405 | 2.196  ±0.779 | 1.882  ±1.636 | 2.094  ±0.546 |  |  |  |  |  |
| PC ae C34:2 | 1.062  ±0.407 | 1.352  ±0.578 | 1.206  ±0.641 | 1.268  ±0.219 |  |  |  |  |  |
| PC ae C34:3 | 0.416  ±0.128 | 0.411  ±0.300 | 0.434  ±0.388 | 0.388  ±0.106 |  |  |  |  |  |
| PC ae C36:1 | 1.236  ±0.336 | 1.640  ±0.366 | 1.318  ±0.620 | 1.712  ±0.554 | +75.4  (0.034) |  |  |  |  |
| PC ae C36:2 | 0.836  ±0.201 | 1.055  ±0.395 | 0.831  ±0.722 | 1.029  ±0.346 |  |  |  |  |  |
| PC ae C36:3 | 0.406  ±0.097 | 0.384  ±0.401 | 0.434  ±0.378 | 0.457  ±0.166 |  |  |  |  |  |
| PC ae C36:4 | 1.506  ±0.386 | 1.627  ±1.071 | 1.551  ±1.355 | 1.871  ±0.527 |  |  |  |  |  |
| PC ae C36:5 | 3.846  ±1.948 | 4.131  ±1.261 | 3.678  ±1.386 | 4.415  ±1.478 |  |  |  |  |  |
| PC ae C38:2 | 0.399  ±0.135 | 0.533  ±0.322 | 0.476  ±0.174 | 0.692  ±0.285 |  | +73.4  (0.011) |  | +45.4  (0.009) |  |
| PC ae C38:3 | 0.522  ±0.176 | 0.739  ±0.457 | 0.555  ±0.224 | 0.861  ±0.189 |  | +64.9  (0.018) |  | +55.1  (0.033) |  |
| PC ae C38:4 | 1.537  ±0.418 | 1.938  ±0.775 | 1.739  ±0.967 | 2.254  ±0.610 |  | +46.6  (0.038) |  |  |  |
| PC ae C38:5 | 2.670  ±1.154 | 2.933  ±0.844 | 2.719  ±1.626 | 3.372  ±0.708 |  |  |  |  |  |
| PC ae C38:6 | 1.087  ±0.546 | 1.083  ±0.354 | 1.152  ±0.413 | 1.347  ±0.265 |  |  |  |  |  |
| PC ae C40:2 | 0.341  ±0.080 | 0.477  ±0.242 | 0.341  ±0.118 | 0.494  ±0.130 |  | +44.9  (0.036) |  | +44.9  (0.045) |  |
| PC ae C40:3 | 0.262  ±0.074 | 0.359  ±0.218 | 0.270  ±0.116 | 0.378  ±0.125 |  | +44.3  (0.047) |  |  |  |
| PC ae C40:4 | 0.212  ±0.070 | 0.266  ±0.122 | 0.247  ±0.125 | 0.317  ±0.068 |  |  |  |  |  |
| PC ae C40:5 | 0.375  ±0.120 | 0.429  ±0.168 | 0.392  ±0.241 | 0.576  ±0.122 |  | +53.6  (0.046) |  |  |  |
| PC ae C40:6 | 0.353  ±0.141 | 0.381  ±0.159 | 0.354  ±0.183 | 0.543  ±0.124 |  |  |  |  |  |
| PC ae C42:2 | 0.050  ±0.015 | 0.056  ±0.031 | 0.053  ±0.070 | 0.076  ±0.028 |  | +52.0  (0.033) |  |  |  |
| PC ae C42:3 | 0.048  ±0.013 | 0.036  ±0.029 | 0.048  ±0.078 | 0.065  ±0.033 |  |  |  |  |  |
| PC ae C44:5 | 0.104  ±0.029 | 0.092  ±0.144 | 0.077  ±0.094 | 0.116  ±0.061 |  |  |  |  |  |
| **Hexoses** | | | | |  |  |  |  |  |
| H1 | 370.3  ±164.9 | 385.9  ±165.1 | 400.4  ±260.2 | 238.1  ±272.3 |  |  |  |  |  |
| **Amino Acids** | | | | |  |  |  |  |  |
| Gln | 51.76  ±28.46 | 52.16  ±9.12 | 66.38  ±67.36 | 65.99  ±27.18 |  |  |  |  |  |
| Gly | 35.60  ±16.98 | 36.35  ±11.85 | 41.64  ±47.27 | 39.11  ±83.63 |  |  |  |  |  |
| His | 4.691  ±34.67 | 5.346  ±85.4 | 4.825  ±4.77 | 6.224  ±8.90 |  |  |  |  |  |
| Phe | 7.483  ±2.261 | 9.070  ±1.602 | 8.933  ±9.361 | 11.599  ±3.707 |  |  |  |  |  |
| Pro | 16.26  ±4.47 | 19.52  ±3.26 | 19.79  ±20.46 | 21.17  ±7.82 |  |  |  |  |  |
| Ser | 11.80  ±5.35 | 12.03  ±1.54 | 12.08  ±27.10 | 14.17  ±42.54 |  |  |  |  |  |
| Thr | 5.129  ±3.20 | 6.267  ±1.344 | 6.839  ±12.61 | 7.250  ±7.17 |  |  |  |  |  |
| Tyr | 11.13  ±2.44 | 12.11  ±2.07 | 11.67  ±12.60 | 15.05  ±5.03 |  |  |  |  |  |
| Val | 11.68  ±4.96 | 12.30  ±4.15 | 15.51  ±17.24 | 16.74  ±10.15 |  |  |  |  |  |
| xLeu | 37.86  ±19.07 | 40.65  ±7.81 | 49.46  ±50.40 | 48.64  ±14.91 |  |  |  |  |  |
| **Acylcarnitines** | | | | |  |  |  |  |  |
| C2 | 1.887  ±0.862 | 1.123  ±0.995 | 1.343  ±0.961 | 1.140  ±0.485 |  |  |  |  |  |
| C5 | 0.038  ±0.025 | 0.053  ±0.014 | 0.038  ±0.014 | 0.037  ±0.015 |  |  |  |  |  |
| **Ceramides** | | | | |  |  |  |  |  |
| N-C14:0-Cer | 0.021  ±0.007 | 0.021  ±0.003 | 0.022  ±0.005 | 0.025  ±0.004 |  |  |  |  |  |
| N-C16:0-Cer | 0.380  ±0.140 | 0.286  ±0.510 | 0.366  ±0.190 | 0.378  ±0.080 |  |  |  |  |  |
| N-C24:1-Cer | 0.240  ±0.136 | 0.195±0.075 | 0.222  ±0.176 | 0.259  ±0.059 |  |  |  |  |  |
| N-C24:0-Cer | 0.220  ±0.095 | 0.091  ±0.047 | 0.130  ±0.104 | 0.149  ±0.036 |  |  |  |  | +63.7  (0.042) |

Table A in S1 File summarizes the absolute concentrations of individual metabolites (2^nd^ to 5^th^ row from left), by metabolite class. Only metabolites above the lower level of detection and metabolites which differed significantly between at least two groups (ANOVA analyses and multiple t-tests (non-adjusted for maximum sensitivity)) are listed. Values are given as median ± SD (in µM). C, control; BAV-A, bicuspid aortic valve-associated thoracic aneurysm; TAV-A, tricuspid aortic valve-associated thoracic aneurysm; TAV-Diss, tricuspid aortic valve-associated thoracic dissection. In the right side of the table the difference between groups is given in %; the corresponding p-values are indicated. “+” prior to the values (in %) indicate that the first group shows higher concentrations compared to the second group. B-C, BAV-A versus C; D-C, TAV-Diss versus C; D-T, TAV-Diss versus TAV-A. Comparisons: TAV-A versus C is not given, because no significant differences between groups were observed. Empty fields indicate the absence of a significant difference.

**Figure A in S1 File -** **Total sphingomyelin concentrations in non-diabetic controls and different ATAA samples**




Figure A in S1 File shows the disease group-specific concentration of the sum of metabolites for sphingomyelins. The sums given include only those metabolites per class that were included in the analysis (for details see S1-Table). Patients suffering from diabetes were excluded from the analysis. C, control (n = 4); BAV-A, bicuspid aortic valve-associated aneurysm (n = 9); TAV-A, tricuspid aortic valve-associated aneurysm (n = 11); TAV-Diss, tricuspid aortic valve-associated dissection (n = 6). Dots represent median values per patient sample; black line indicates the groups mean value. Asterisks indicate significant differences between groups (ANOVA, Bonferroni adjusted) *, p<0.05.

**Fig B in S1 File -** **Hierarchical cluster analysis of total metabolite concentrations**


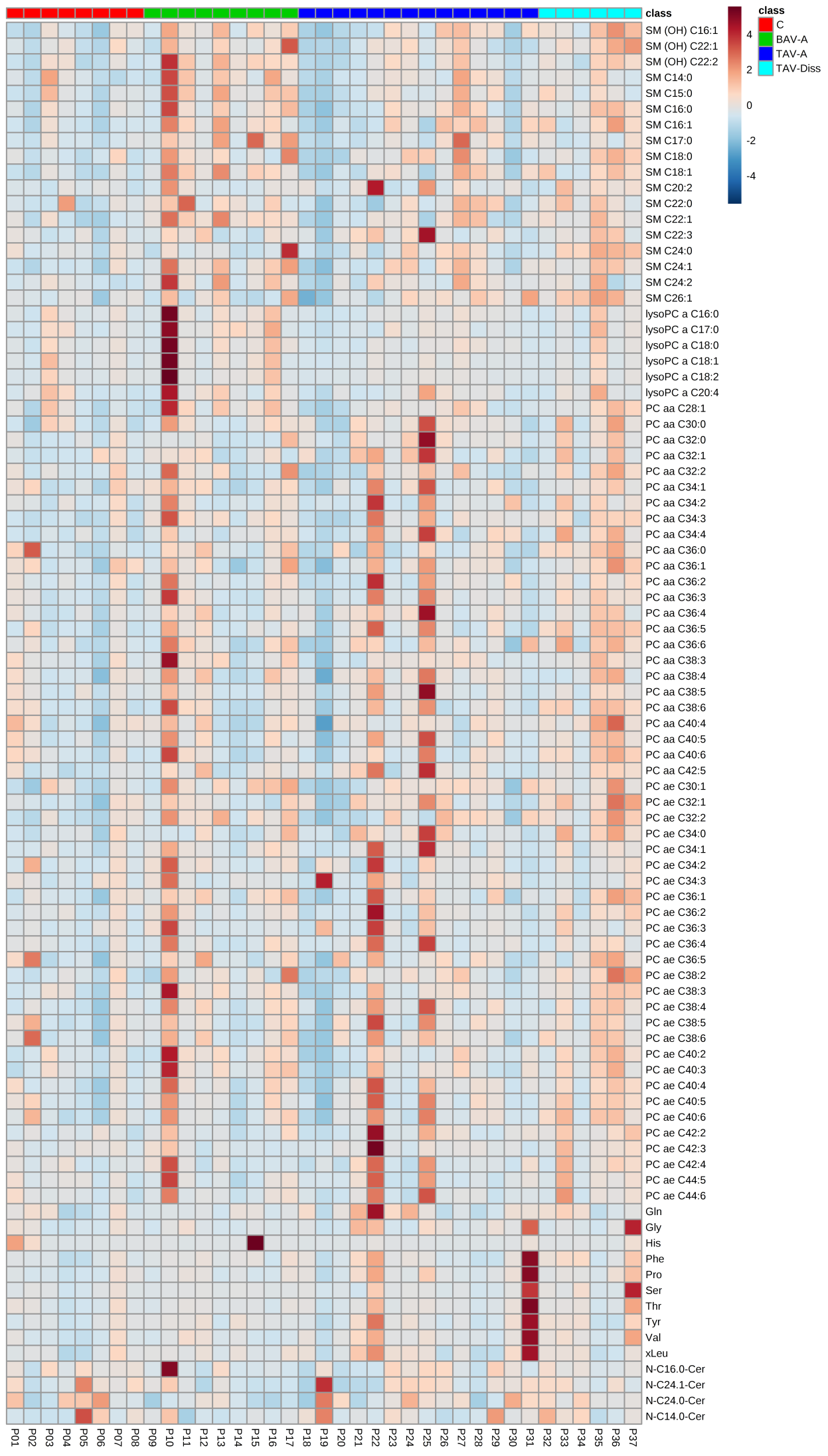


Figure B in S1 File shows the distribution of total metabolite concentrations (i.e. sphingomyelins, glycerophospholipids, amino acids and ceramides) per patient tissue sample by hierarchical cluster analysis. Metabolite identity is indicted on the right side. Colors indicate compound concentrations. Color scale bar (low concentration (dark blue) to high concentration (dark red) and group color code are given in the upper right. C, control; BAV-A, bicuspid aortic valve-associated thoracic aneurysm; TAV-A, tricuspid aortic valve-associated thoracic aneurysm; TAV-Diss, tricuspid aortic valve-associated thoracic dissection.
